# Intermittent parathyroid hormone (1-34) ameliorates periodontitis via Treg-dependent immunomodulation of the Treg/Th17 axis

**DOI:** 10.3389/fimmu.2026.1862703

**Published:** 2026-07-30

**Authors:** Cheng Zhang, Cong Deng, Linkun Zhang

**Affiliations:** 1 Department of Orthodontics, Tianjin Stomatological Hospital, School of Medicine, Nankai University, Tianjin, China; 2 Tianjin Key Laboratory of Oral and Maxillofacial Function Reconstruction & Stomatology Institute of Nankai University, Tianjin, China

**Keywords:** alveolar bone loss, parathyroid hormone, periodontitis, regulatory T cell, teriparatide, Th17 cell

## Abstract

**Objectives:**

The intermittent administration of parathyroid hormone 1-34 (iPTH) is a bone anabolic therapy with potential for periodontitis treatment, but its immunomodulatory mechanisms remain unclear. This study investigated whether iPTH ameliorates periodontitis by modulating the regulatory T cell (Treg)/T helper cell (Th17) balance.

**Methods:**

Experimental periodontitis was induced in mice by ligature. Mice were assigned to control, periodontitis, or iPTH-treated periodontitis groups. Alveolar bone loss was analyzed by micro computed tomography. Bone turnover, cytokines and immune cells were assessed by histology, immunohistochemistry, immunofluorescence, quantitative polymerase chain reaction, and flow cytometry. The role of Tregs was verified via anti-CD25 antibody-mediated depletion.

**Results:**

iPTH treatment significantly attenuated alveolar bone loss, improved trabecular microarchitecture, enhanced osteoblast activity, and suppressed osteoclastogenesis. Besides, iPTH suppressed the inflammatory level, downregulating interleukin (IL)-6, IL-17a, receptor activator of nuclear factor-κB ligand (RANKL), while upregulating IL-10. Crucially, iPTH shifted the Treg/Th17 functional axis toward an anti-inflammatory state, increasing Treg proportions in cervical lymph nodes and forkhead box protein P3 (FOXP3) level while simultaneously suppressing IL-17 in gingiva. Furthermore, iPTH reduced the periodontitis-enhanced spatial proximity between FOXP3 and IL-17. Depletion of Tregs impaired the bone-protective effects of iPTH.

**Conclusions:**

iPTH ameliorates periodontitis-induced alveolar bone loss through a Treg-dependent mechanism. This identifies the immunomodulation of the Treg/Th17 axis as a pivotal mechanism underlying iPTH’s efficacy, highlighting its potential as a host-modulating therapy for periodontitis.

## 1 Introduction

Periodontitis is a prevalent chronic inflammatory disease characterized by the progressive destruction of periodontal supporting tissues, leading to tooth mobility and eventual tooth loss (1). The pathogenesis of periodontitis involves a dysregulated host immune response to periodontal pathogens, resulting in excessive osteoclastogenesis and impaired osteogenesis, leading to tissue damage (2). Although conventional treatments such as scaling and root planning can mitigate inflammation, they are not always effective in managing periodontitis due to anatomical and/or systemic factors, and their bone regenerative potential is limited. Therefore, adjunctive therapies are considered to enhance clinical outcomes by regulating host response and promoting bone regeneration (3, 4).

Intermittent parathyroid hormone (iPTH) administration is a well-established approach for osteoporosis treatment, most notably the recombinant human PTH (1-34) fragment, teriparatide (5). Emerging evidence suggests that iPTH may also exert protective effects in inflammatory bone diseases including periodontitis (6–9). However, the underlying mechanisms, especially its immunomodulatory effects, remain poorly understood.

CD4^+^ T lymphocytes play a vital role in bone homeostasis and inflammatory responses (10). Specifically, the balance between regulatory T cells (Treg) and T helper 17 cells (Th17) is critical in maintaining immune homeostasis and bone metabolism (11). Tregs, characterized by the expression of Forkhead box protein P3 (FOXP3) and interleukin (IL)-10, suppress immune responses and protect against bone erosion (12), whereas Th17 cells, marked by the expression of retinoic acid receptor-related orphan receptor gamma t (RORγt), promote inflammation and osteoclastogenesis via IL-17 secretion. An imbalance in the Treg/Th17 ratio has been implicated in the pathogenesis of periodontitis (13, 14).

Notably, iPTH has been reported to expand Treg populations in human and mice (15, 16). However, whether iPTH modulates the Treg/Th17 axis in the context of periodontitis to ameliorate bone loss remains unexplored.

Therefore, we hypothesized that iPTH alleviates alveolar bone loss in periodontitis by restoring the Treg/Th17 axis. To test this, we established a murine model of experimental periodontitis and evaluated the effects of iPTH on alveolar bone mass, osteoclast activity, osteogenic function, inflammatory level and Treg/Th17 variation in gingival tissues and submandibular lymph nodes. Furthermore, we depleted Tregs using anti-CD25 antibody to validate their role in iPTH-mediated bone protection. Our study deepens researchers’ understanding of the immunopharmacological effects of iPTH, providing a theoretical basis for its novel use as a host-regulating adjuvant therapy for periodontitis.

## 2 Materials and methods

### 2.1 Experimental animals

All animal experiments were conducted in accordance with Animal Research: Reporting of *In Vivo* Experiments (ARRIVE) 2.0 guidelines and regulations and were approved by the Medical Ethics Committee of Tianjin Stomatological Hospital (PA2023-S-006). 8-week-old female C57BL/6J mice (Beijing Vital River Laboratory Animal Technology Co. Ltd., China) were used. All mice were specific pathogen-free (SPF) and immunocompetent, genetically wild-type with no prior procedures. They were housed under SPF conditions in individually ventilated cages with aspen chip bedding and had free access to standard rodent chow and autoclaved water in a temperature-controlled room (22 ± 1 °C, 50–60% relative humidity) under a 12/12-hour light/dark cycle. Upon arrival, all mice were allowed to acclimate to the animal facility for 7 days prior to the start of any experimental procedures.

### 2.2 Study design

#### 2.2.1 Grouping

This *in-vivo* study comprised two sequential stages.

Stage I: Mice were randomly and evenly assigned to one of three groups: Control (CON): Healthy mice receiving daily injections of normal saline (the vehicle of PTH). Periodontitis (PD): Mice with ligature-induced periodontitis. iPTH+PD: Mice with ligature-induced periodontitis receiving daily iPTH injections.

Stage II: Mice were first randomly assigned to receive either CD25 neutralizing antibody (anti-CD25) or a rat isotype IgG control (IC). Each of these two cohorts was then further subdivided into the same three experimental groups as in Stage I (CON, PD, and iPTH+PD), resulting in a total of six groups.

The experimental unit was the single animal.

#### 2.2.2 Sample size

In Stage I, there were 6 mice in each group, the total number of mice in each experiment is 18. In Stage II, there were 12 mice in each group, the total number of mice in each experiment is 72.

The sample size for this experiment was determined according to previous literature (15, 17–20) and sensitivity power analysis was conducted using G*Power to ensure the validity of the statistical conclusions (α=0.05, power(1–β)=0.80) based on the primary outcome (proportion of Treg among CD4^+^ T cells in cervical lymph nodes measured by flow cytometry).

#### 2.2.3 Inclusion and exclusion criteria

Inclusion criteria: 8-week-old female C57BL/6J mice, with body weight between 18-22g. Exclusion criteria: (1) Any mouse that lost the ligature prematurely during the modeling period was excluded from the final analysis, as the periodontitis induction would be considered incomplete. (2) Any mouse that developed post-operative complications (e.g., severe infection, self-mutilation) or exhibited signs of severe illness (e.g., lethargy, hunched posture, rough hair coat) or had visible injuries or experienced a body weight loss exceeding 15% of its initial weight at any point during the study was to be humanely euthanized and excluded from analysis for animal welfare reasons.

#### 2.2.4 Randomization

A randomization sequence was used to allocate mice to all experimental groups. The randomization sequence was generated using the List Randomizer (https://www.random.org/lists/). Animal procedures were performed in a randomized order daily and cage positions on the rack were re-randomized daily to minimize potential confounders.

#### 2.2.5 Blinding

The personnels performing the animal treatments were aware of the group allocation, while the researcher who analysis the data was blinded to the groups.

#### 2.2.6 Outcome measures

The parameters assessed include: the vertical distance between cementoenamel junction and alveolar bone crest (CEJ-ABC distance); bone mineral density (BMD); trabecular separation (Tb.Sp); trabecular number (Tb.N); trabecular thickness (Tb.Th); bone volume fraction (BV/TV); protein level of FOXP3, IL-17 and bone sialoprotein (BSP); the number of osteoclasts; the gene expression level of *Il6, Il17a, Il10, Tnfsf11*, and *Tnfrsf11b*; the Pearson’s correlation coefficient (PCC) and Manders’ colocalization coefficient M1 of FOXP3 and IL-17; the proportion of Treg and Th17 among CD4^+^ T cells and FOXP3^+^RORγt^+^ cells among Tregs in cervical lymph nodes (CLNs). The primary outcome of this study was the proportion of Treg among CD4^+^ T cells in CLNs measured by flow cytometry. Secondary outcomes were CEJ-ABC distance, BMD, Tb.Sp, Tb.N, Tb.Th and BV/TV measured by micro computed tomography (micro-CT); protein level of FOXP3, IL-17 and BSP measured by immunohistochemistry (IHC), the number of osteoclasts measured by tartrate-resistant acid phosphatase (TRAP) staining; the gene expression level of *Il6, Il17a, Il10, Tnfsf11*, and *Tnfrsf11b* measured by quantitative polymerase chain reaction (qPCR); the Pearson’s correlation coefficient (PCC) and the Manders’ colocalization coefficient (MCC)-M1 of FOXP3 and IL-17 calculated by Immunofluorescence; the proportion of Th17 among CD4^+^ T cells and FOXP3^+^RORγt^+^ cells among Tregs in CLNs measured by flow cytometry.

### 2.3 Periodontitis model establishment

Mice were anesthetized using isoflurane inhalation. Anesthesia was induced with 2.5% isoflurane and maintained with 1.5% isoflurane using an isoflurane vaporizer. Periodontitis model was established in the PD and iPTH+PD groups by ligating a 5–0 silk suture around the cervical region of the left maxillary second molars without causing damage to the periodontal tissues according to literature (17). The ligatures were inspected daily, if ligature loss happened, the mouse was excluded. Mice in the CON group received no ligation.

### 2.4 iPTH administration

For iPTH administration, human PTH (1-34) (HY-P4821, MedChemExpress, USA) was dissolved in normal saline and administered via subcutaneous injection in the dorsal region at a dosage of 40 μg/kg body weight daily since the day of PD establishment, with a 24-hour interval between each injection, a common and generally adopted route of administration according to literatures (21). Mice in the CON and PD groups received equivalent volumes of normal saline vehicle.

Twenty-four hours after the final injection on day 14, all mice were euthanized by carbon dioxide (CO_2_) inhalation. CO_2_ was delivered from a compressed gas cylinder into an airtight chamber at a displacement rate of 30% of the chamber volume per minute to minimize distress. After the mice lost consciousness, the CO_2_ flow was maintained for at least 5 minutes following respiratory arrest. Subsequently, euthanasia was confirmed by cervical dislocation.

### 2.5 *In vivo* Treg depletion

Treg depletion was achieved by subcutaneous injection of anti-mouse CD25 antibody (PC-61.5.3) (A2107, Selleck, USA) at 250μg per mouse on the day of periodontitis induction and iPTH injection according to literature (22). Rat IgG1 isotype control (A2119, Selleck, USA) was used as negative control.

### 2.6 Micro-CT analysis

**Figure 1:**
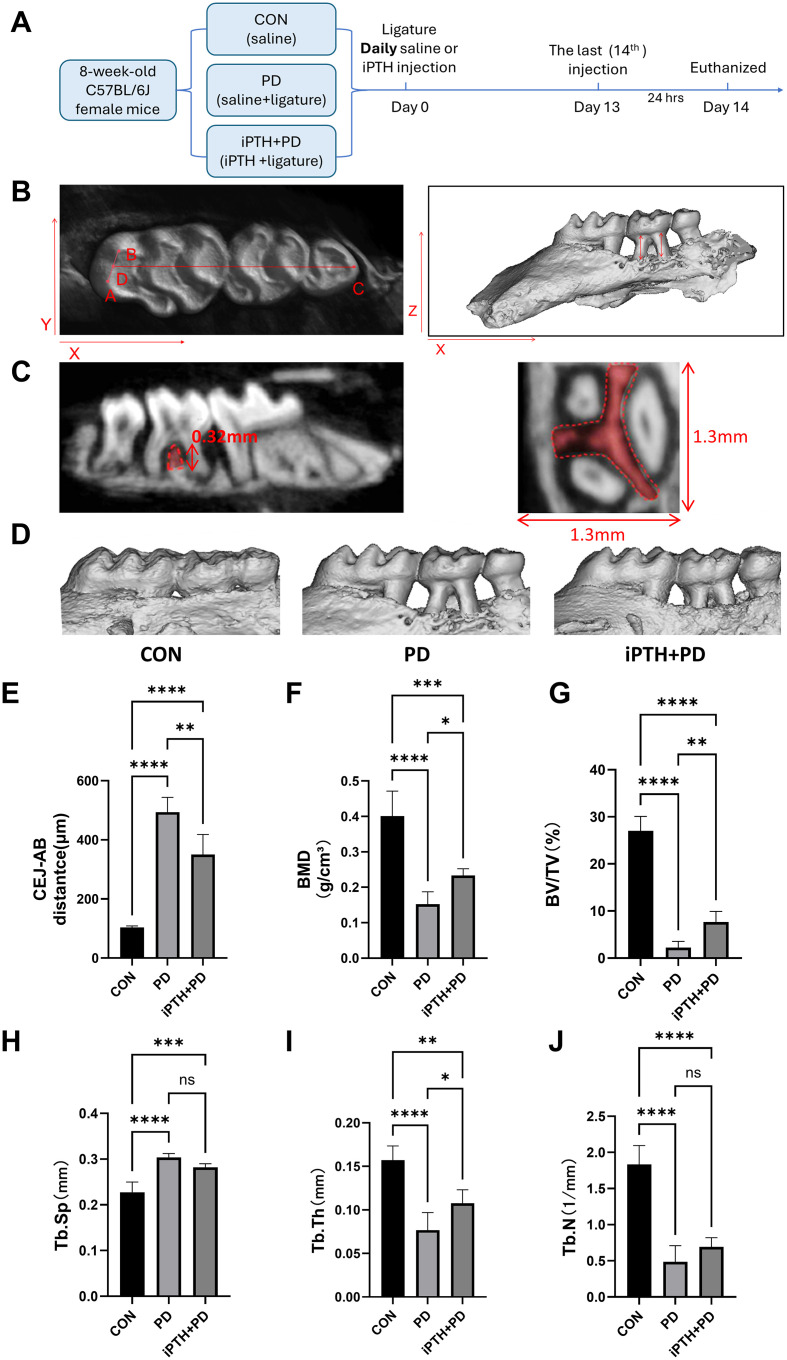
Systemic intermittent parathyroid hormone (1-34) (iPTH) treatment alleviates periodontitis-induced alveolar bone loss in mice. **(A)** Schematic timeline of the experimental design. **(B)** Method for specimen orientation and measuring the cementoenamel junction (CEJ) -alveolar bone crest (ABC) distance on micro computed tomography (micro-CT) 3D reconstructions. **(C)** Definition of the region of interest (ROI) for micro-CT trabecular morphometry analysis. **(D)** Representative buccal-view micro-CT images of alveolar bone from the control (CON), periodontitis (PD), and iPTH-treated periodontitis (iPTH+PD) groups. **(E)** Quantitative analysis of CEJ-ABC distance, **(F)** bone mineral density (BMD), **(G)** bone volume fraction (BV/TV), **(H)** trabecular separation (Tb.Sp), **(I)** trabecular thickness (Tb.Th), and **(J)** trabecular number (Tb.N) (*n* = 5). Data are presented as mean ± SD. Significance was determined by one-way analysis of variance (ANOVA) with Tukey’s *post hoc* test; ns, not significant, *p<0.05, **p<0.01, ***p<0.001, ****p<0.0001.

The maxillae were harvested and fixed in 4% paraformaldehyde at 4 °C for 24 h and preserved in 0.5% paraformaldehyde at 4 °C. Micro-CT scanning (Skyscan1276, Bruker, Germany) with an X-ray tube voltage of 70kVp, current of 200μA, and an isotropic voxel size of 8μm was performed. To quantify the height of alveolar bone loss, 3D images of each specimen were reconstructed using Mimics Medical 20.0 (Materialise, Belgium). The spatial position orientation of the specimen was performed as below (**Figure 1B**): the highest point of the mesiolingual cusp of the left first molar in the mouse was designated as point A, the highest point of the mesiobuccal cusp of the first molar as point B, and the distal cusp of the third molar as point C. The occlusal plane (X-Y plane) was determined by points A, B, and C. The midpoint of A and B was taken as point D, and line segment CD was set parallel to the X-axis. The Z-axis was perpendicular to the occlusal plane, thereby establishing a three-dimensional coordinate system. The measurement site was the midpoint between the mesial and distal buccal roots of the second molar. Using the 3D measurement tool in Mimics, CEJ-ABC distance was measured along a line perpendicular to the occlusal plane (**Figure 1B**). The average of the measured values was taken as the final CEJ-ABC distance for this sample. For alveolar bone quality analysis, a cubic region of interest (ROI) (1.3 mm × 1.3 mm × 0.32 mm) was defined centered on the furcation area of the second molar. Teeth and cortical bone were manually excluded to isolate the trabecular bone within the furcation (**Figure 1C**). BMD and trabecular morphometric indices including Tb.Sp, Tb.N, Tb.Th, and BV/TV were analyzed using CTAn (CT-Analyser) (Bruker, USA).

### 2.7 Histological analyses

Maxillae were first fixed in 4% paraformaldehyde at 4 °C for 24h, then decalcified in 10% ethylenediaminetetraacetic acid (EDTA) at room temperature for 4 weeks. After being dehydrated using a dehydrator, the tissue is embedded in paraffin, and sectioned along sagittal direction at 4μm for histological staining. After deparaffinization and rehydration, sections were ready for following use.

#### 2.7.1 TRAP staining

TRAP staining was performed using a commercial TRAP kit (D023-1-1, Nanjing Jiancheng Bioengineering Institute, China) according to the manufacturer’s protocol. Osteoclasts, defined as TRAP-positive multinucleated cells (≥3 nuclei) located within the alveolar bone and within 100μm of the periodontal ligament of the maxillary second molar, were quantified.

#### 2.7.2 Immunohistochemistry

Sections underwent antigen retrieval with 0.01M sodium citrate buffer solution (pH=6.0) for 10min at 95 °C. Endogenous peroxidase activity was quenched with 3% H_2_O_2_ for 10min at room temperature. Sections were then blocked with 10% goat serum for 30min at room temperature and incubated overnight at 4 °C with primary antibodies against IL-17 (66148-1-Ig, Proteintech, China), FOXP3 (EPR22102-37, Abcam, UK), and BSP (GTX12155, GeneTex, USA). Immunoreactivity was visualized using a DAB chromogen kit (ZLI-9017, ZSGB-BIO, China), and nuclei were counterstained with hematoxylin. Sections were dehydrated through graded ethanol series (70%, 95%, and 100% ethanol), cleared in xylene, and mounted with neutral mounting medium. Quantitative analysis of staining was performed using Image-Pro Plus 6.0 software (Media Cybernetics, USA) by measuring the mean optical density (MOD) of target proteins.

#### 2.7.3 Hematoxylin and eosin staining

Sections were stained with hematoxylin solution for 5 min, rinsed in tap water for 5 min, differentiated in 1% acid alcohol (1% hydrochloric acid in 70% ethanol) for 2–3 s, and then blued in 0.2% ammonia water for 1 min. The sections were subsequently stained with 1% eosin Y solution for 2 min, dehydrated through graded ethanol series (70%, 95%, and 100% ethanol), cleared in xylene, and mounted with neutral mounting medium. All steps were carried out at room temperature.

#### 2.7.4 Immunofluorescence

For immunofluorescence, after antigen retrieval and blocking mentioned in Section 2.7.2, sections were incubated with a mixture of mouse anti-IL-17 (66148-1-Ig, Proteintech, China) and rabbit anti-FOXP3 (EPR22102-37, Abcam, UK) primary antibodies overnight at 4 °C. Subsequently, sections were incubated with PE-conjugated goat anti-rabbit IgG (SA00008-2, Proteintech, China) and CoraLite488-conjugated goat anti-mouse IgG (SA00013-1, Proteintech, China) secondary antibodies for 1 h at 37 °C. Finally, sections were mounted with an antifade mounting medium containing DAPI (P0131, Beyotime, China) and imaged using a Ni-U upright fluorescence microscope (Nikon, Japan). Colocalization analysis was performed using ImageJ software (NIH, Bethesda, MD, USA) with the Coloc 2 plugin. A binary mask was generated by manually setting a threshold on the red channel. The masked regions of morphologically intact red-positive cells or cell clusters were defined as ROIs. For each ROI, the PCC and the MCC-M1 (i.e., the proportion of red signal overlapping with green signal relative to the total red signal within the red-positive area) were calculated using the Coloc 2 plugin. The same threshold parameters were consistently applied across all sections to ensure comparability.

### 2.8 Quantitative polymerase chain reaction

Gingival tissues surrounding the left maxillary molars were meticulously dissected as literature (18), snap-frozen in liquid nitrogen, and stored at -80 °C for subsequent RNA extraction. Gingival tissues from every two mice were randomly pooled as one sample before RNA extraction. Total RNA was extracted from gingival tissues using the TaKaRa MiniBEST Universal RNA Extraction Kit (TaKaRa, Japan). RNA concentration and purity were assessed spectrophotometrically (NanoDrop™ One, Thermo Fisher Scientific, USA), and only samples with an A260/A280 ratio between 1.8 and 2.1 were used. cDNA was synthesized using the PrimeScript™ RT reagent Kit (TaKaRa, Japan). Quantitative PCR was performed using TB Green^®^ Premix Ex Taq™ II (Tli RNaseH Plus) (TaKaRa, Japan) on a LightCycler 480 Real-Time PCR System (Roche, Switzerland). The thermal cycling conditions were as follows: initial denaturation at 95 °C for 2 min; followed by 40 cycles of denaturation at 95 °C for 15 seconds, annealing at 60 °C for 15 seconds, and extension at 72 °C for 1 min. A melt curve analysis was performed at the end of each run to confirm the specificity of the amplification products. All reactions were performed in triplicate for each sample. Gene expression was normalized to *Gapdh* and calculated using the 2^-ΔΔCt^ method. The primer sequences were listed in **Supplementary Materials** (**Supplementary Table 1**).

### 2.9 Flow cytometry

Single-cell suspensions were prepared by crushing submandibular lymph nodes and filtered through a 40-μm cell strainer. Cells were stained with fluorochrome-conjugated monoclonal antibodies: anti-CD4 (RM4.5) (Alexa Fluor 488) (557667, BD biosciences, USA), anti-CD25 (PC61.5) (PE-Cyanine7) (25-0251-82, eBioscience, USA), anti-FOXP3 (FJK-16s) (eFluor™ 660) (50-5773-82, eBioscience, USA), and anti-RORγt (Q31-378) (BD Horizon™ RB705) (BD biosciences, USA). Fixable viability dye eFluorTM 450 (65-0863-14, eBioscience, USA) was used to discriminate dead cells. Anti-CD16/CD32 (2.4G2) (553141, BD biosciences, USA) was used for FcR blocking and surface staining for CD4 and CD25 was performed, at 4°C for 30min. Cells were then fixed and permeabilized using FOXP3/Transcription Factor Staining Buffer Set (00-5523-00, eBioscience, USA) overnight, and intracellularly stained for FOXP3 and RORγt at 4°C for 30min. Data acquisition was performed on a CytoFLEX S flow cytometer (Beckman Coulter, USA), and data were analyzed using FlowJo software. Gating strategy was shown in **Supplementary Materials** (**Supplementary Figure 1**).

### 2.10 Statistical analysis

For normal distribution, data were presented as mean with standard deviation (SD). Statistical significance was determined by one-way or two-way analysis of variance (ANOVA) followed by Tukey’s multiple comparisons test. For nonparametric distribution, data were presented as median with interquartile range (IQR). Statistical significance was determined by Kruskal-Wallis test followed by Dunn’s multiple comparisons test. Statistical analysis was performed using GraphPad Prism 10.6 (GraphPad Software, USA). A *p*-value of ≤ 0.05 was considered statistically significant (*p ≤ 0.05, **p ≤ 0.01, ***p ≤ 0.001, ****p ≤ 0.0001).

## 3 Results

### 3.1 iPTH ameliorates alveolar bone loss of periodontitis in mice

The experimental design timeline was shown in **Figure 1A**. The amount of alveolar bone loss was measured in terms of alveolar bone height and quality. Alveolar bone height was assessed by measuring the CEJ-ABC distance. As shown in **Figures 1D, E**, compared with the CON group, the PD group exhibited a significant increase in the CEJ-ABC distance at day 14 (*p* < 0.0001). However, the iPTH+PD group demonstrated a notable decrease (*p* = 0.0014) in the CEJ-ABC distance relative to the PD group.

The mineral density and the microarchitecture of alveolar bone were also evaluated (**Figures 1F–J**). Compared with the CON group, the PD group exhibited statistically lower BMD, BV/TV, Tb.Th, and Tb.N, along with greater Tb.Sp (all *p* < 0.0001). Moreover, compared to the PD group, the iPTH+PD group showed significant increases in BMD (*p* = 0.0433), BV/TV (*p* = 0.0076), and Tb.Th (*p* = 0.0403), but similar level in Tb.Sp. (*p* = 0.0846) and Tb.N (*p* = 0.3067).

In all the parameters mentioned above, the data in the iPTH+PD group were significantly differs from the CON group (all *p* < 0.01).

These results indicated that the periodontitis mouse model was successfully established, and iPTH injection partially ameliorated alveolar bone loss of bone height and trabecular quality in this model.

### 3.2 iPTH promotes osteoblast activity and inhibits osteoclast activity at periodontitis lesion area

To further evaluate bone formation and resorption activities along with tissue morphology, TRAP staining and immunohistochemical staining for BSP, as well as HE staining was performed.

**Figure 2:**
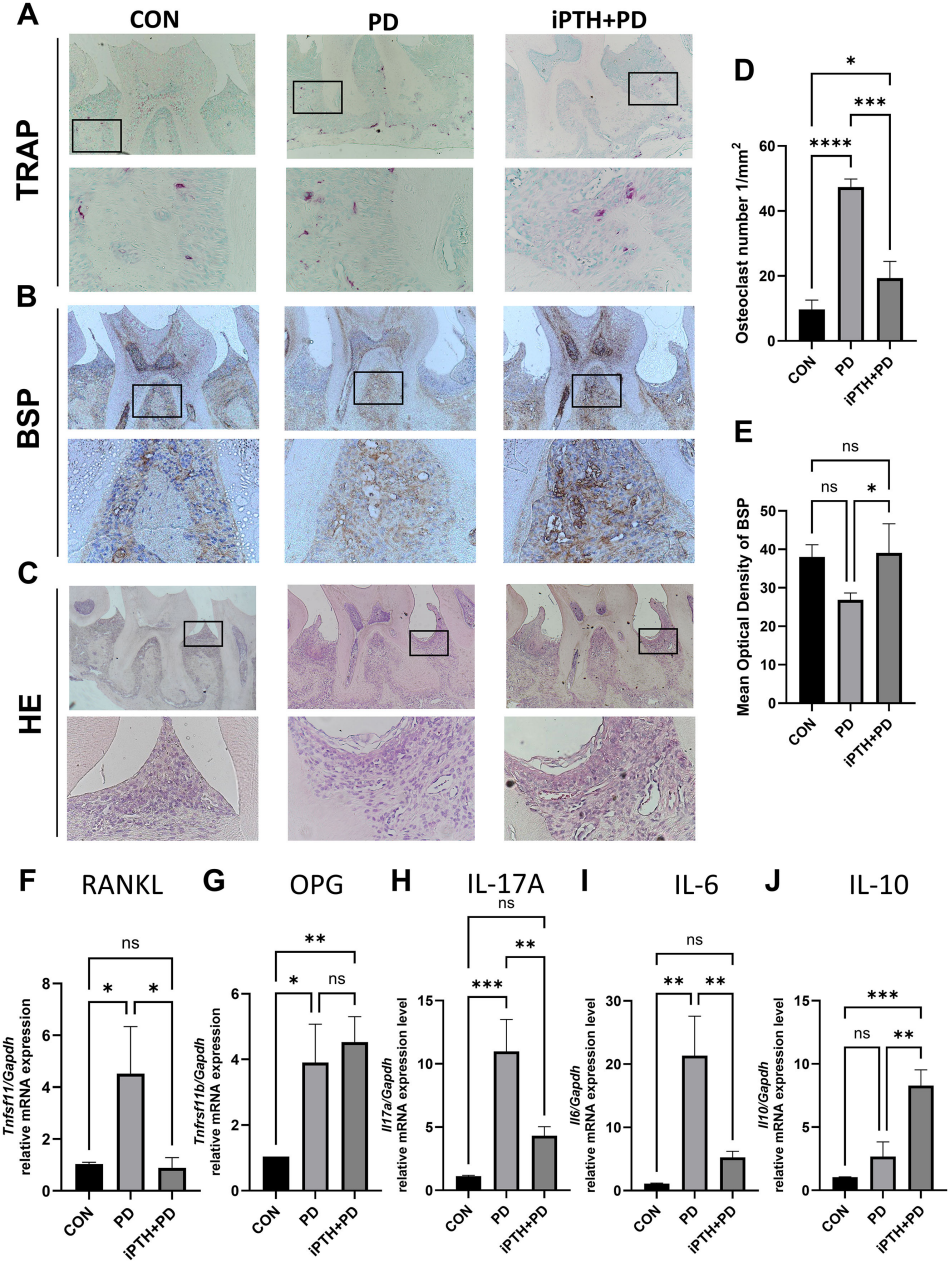
Systemic iPTH injection modulates alveolar bone remodeling and the gingival inflammatory microenvironment in periodontitis. **(A)** Representative images of tartrate-resistant acid phosphatase (TRAP)-stained osteoclasts and **(D)** its quantitative analysis. **(B)** Representative images of bone sialoprotein (BSP) immunohistochemical staining and **(E)** its quantitative analysis. **(C)** Representative hematoxylin and eosin (HE) -stained sections of the periodontal tissue. **(F–J)** mRNA expression levels of *Tnfsf11*, *Tnfrsf11b*, *Il17a, Il6, Il10* in the gingiva from the periodontitis site. Data are presented as mean ± SD (*n* = 3). Significance was determined by one-way ANOVA with Tukey’s *post hoc* test. ns, not significant; *p<0.05, **p<0.01, ***p<0.001, ****p<0.0001.

As shown in **Figures 2A, D**, the number of osteoclasts was markedly higher in the PD group than in the CON group (*p* < 0.0001). Conversely, the iPTH+PD group showed a significant reduction in osteoclast number compared to the PD group (*p* = 0.0002), although exhibited an increasing trend compared to the CON group (*p* = 0.0424).

Regarding BSP, a marker for bone formation, as shown in **Figures 2B, E**, the MOD of BSP showed a downward trend in the PD group compared to the CON but with no significance (*p=*0.0681). In contrast, the iPTH+PD group displayed an increase relative to the PD group (*p=*0.0493), but no significant change compared to the CON group (*p* = 0.9636).

HE staining revealed that in the CON group, the sulcular epithelium of the second molars was continuous and intact, with orderly arranged fibroblasts. In contrast, in the PD group and the iPTH+PD group, the sulcular epithelium was disrupted, superficial keratinized cells were shed, and the collagen fibers in the lamina propria were disorganized with inflammatory cells aggregation (**Figure 2C**).

These results showed that under periodontitis conditions in mice, bone resorption was enhanced, while iPTH administration promoted bone formation and inhibited bone resorption within the 14-day experimental period.

### 3.3 iPTH suppresses inflammatory response in gingiva at periodontitis lesion area

Given the correlation between bone turnover and inflammation, we next examined the expression of genes related to bone metabolism and inflammatory cytokines in gingival tissues.

Quantitative PCR analysis revealed that the expression of *Tnfsf11*, which encodes receptor activator of nuclear factor-κB ligand (RANKL), was significantly up-regulated in the PD group compared to the CON group (*p=*0.0171), but was markedly down-regulated in the iPTH+PD group relative to the PD group (*p=*0.0142) (**Figure 2F**). In Contrast, *Tnfrsf11b* (encoding osteoprotegerin, OPG) was also significantly elevated in the PD group compared to the CON group (*p=*0.0118); however, no significant difference was observed between the iPTH+PD and PD groups (*p=*0.6348) (**Figure 2G**). Besides, compared with the CON group, the iPTH+PD group showed no change in *Tnfsf11* level (*p=*0.9848) but a significant elevation in *Tnfrsf11b* level (*p=*0.0046).

The expression levels of the pro-inflammatory cytokines *Il17a* and *Il6* were pronouncedly higher in the PD group than in the CON group (*p=*0.0005 and *p=*0.0012, respectively). Conversely, their expression was strongly suppressed in the iPTH+PD group compared to the PD group (*p=*0.0041 and *p=*0.0040, respectively). Meanwhile, the gene level of both cytokines showed no statistical difference between the CON and the iPTH+PD group (*p* = 0.0917 for *Il17a* and *p* = 0.4010 for *Il6*) (**Figures 2H, I**).

As for the anti-inflammatory cytokine *Il10*, its expression was slightly increased in the PD group compared to the CON group without statistical significance (*p=*0.1893). Nevertheless, *Il10* expression was notably elevated in the iPTH+PD group compared with the PD group (*p* = 0.0010) and the CON group (*p* = 0.0003) (**Figure 2J**).

These results indicated that osteoclastogenesis and inflammatory responses were enhanced during periodontitis, whereas iPTH treatment suppressed these effects.

### 3.4 iPTH modulates the level and distribution of FOXP3 and IL-17 at periodontitis site

**Figure 3:**
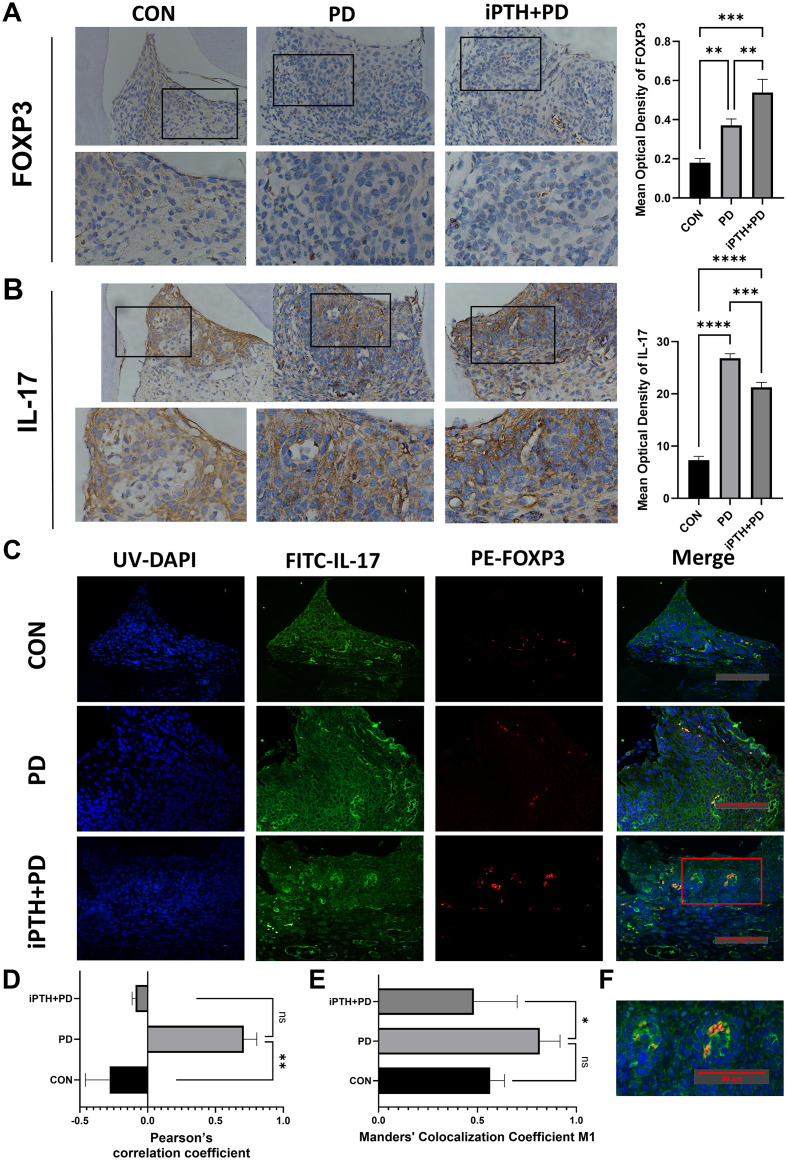
Systemic iPTH injection modulates the expression and spatial relationship of forkhead box protein P3 (FOXP3) and interleukin (IL)-17 in gingival tissues in periodontitis model. Representative images and quantitative analysis of immunohistochemical staining for **(A)** FOXP3 and **(B)** IL-17. **(C)** Representative immunofluorescence images showing FOXP3 (red) and IL-17 (green) expression. **(D)** Pearson’s correlation coefficient and **(E)** Manders’ Colocalization Coefficients M1 of FOXP3 and IL-17 colocalization analysis. **(F)** High-magnification (400×) view of the merged channel from the iPTH+PD group. Data are presented as mean ± SD for one-way ANOVA test or median ± IQR for Kruskal-Wallis test. Significance was determined by one-way ANOVA with Tukey’s *post hoc* test for immunohistochemistry (*n* = 3), and by Kruskal-Wallis test for co-localization analysis (*n* = 3). ns, not significant; *p<0.05, **p<0.01, ***p<0.001, ****p<0.0001.

Given that the above data showed iPTH significantly suppressed the expression of Th17-related cytokine IL-17A and Treg-related IL-10, we next measured the protein levels of FOXP3 and IL-17 in the gingiva by IHC, serving as indicators of local suppressive signaling and IL-17-driven inflammation, respectively. The result showed a significant increase in MOD of FOXP3 in the PD group compared to the CON group (*p=*0.005); a further significant increase was observed in the iPTH+PD group compared to the PD group (*p=*0.0092) (**Figure 3A**). Meanwhile, the MOD of IL-17 in the PD group was markedly higher than the CON group (*p<*0.0001), whereas in the iPTH+PD group, its level decreased with statistical difference (*p=*0.0005), though still markedly increased compared with the CON group (*p* < 0.0001) (**Figure 3B**).

Immunofluorescence staining exhibited the spatial proximity between FOXP3 (red) and IL-17 (green) (**Figures 3C, F**). The ROI was defined as the red-positive area, i.e. the FOXP3^+^ area. The PCC and the MCC-M1 were calculated (**Figures 3D, E**). The colocalization analysis showed that in the CON group, the PCC was -0.1475 ± 0.4610 and the M1 value was 0.4616 ± 0.2409, indicating a weak negative correlation and only moderate spatial overlap between the two proteins under normal conditions. In the PD group, the PCC significantly increased to 0.4763 ± 0.4092 (*p=*0.0086) and the M1 value elevated to 0.7378 ± 0.2312 though without statistical significance (*p=*0.0646), suggesting that periodontitis induced aberrant co-aggregation and intensity synchronization of the two proteins. Following iPTH treatment, the PCC decreased to 0.0200 ± 0.2717 without significance (*p=*0.1927) and the M1 value markedly declined to 0.4470 ± 0.2877 (*p=*0.0412), indicating a downward trend of co-localization.

These results suggested that iPTH up-regulated FOXP3 level as well as reversed the elevation of IL-17 and the colocalization of FOXP3 and IL-17 induced by periodontitis at the lesion site.

### 3.5 Treg proportion in CLNs was raised by iPTH in periodontitis

**Figure 4:**
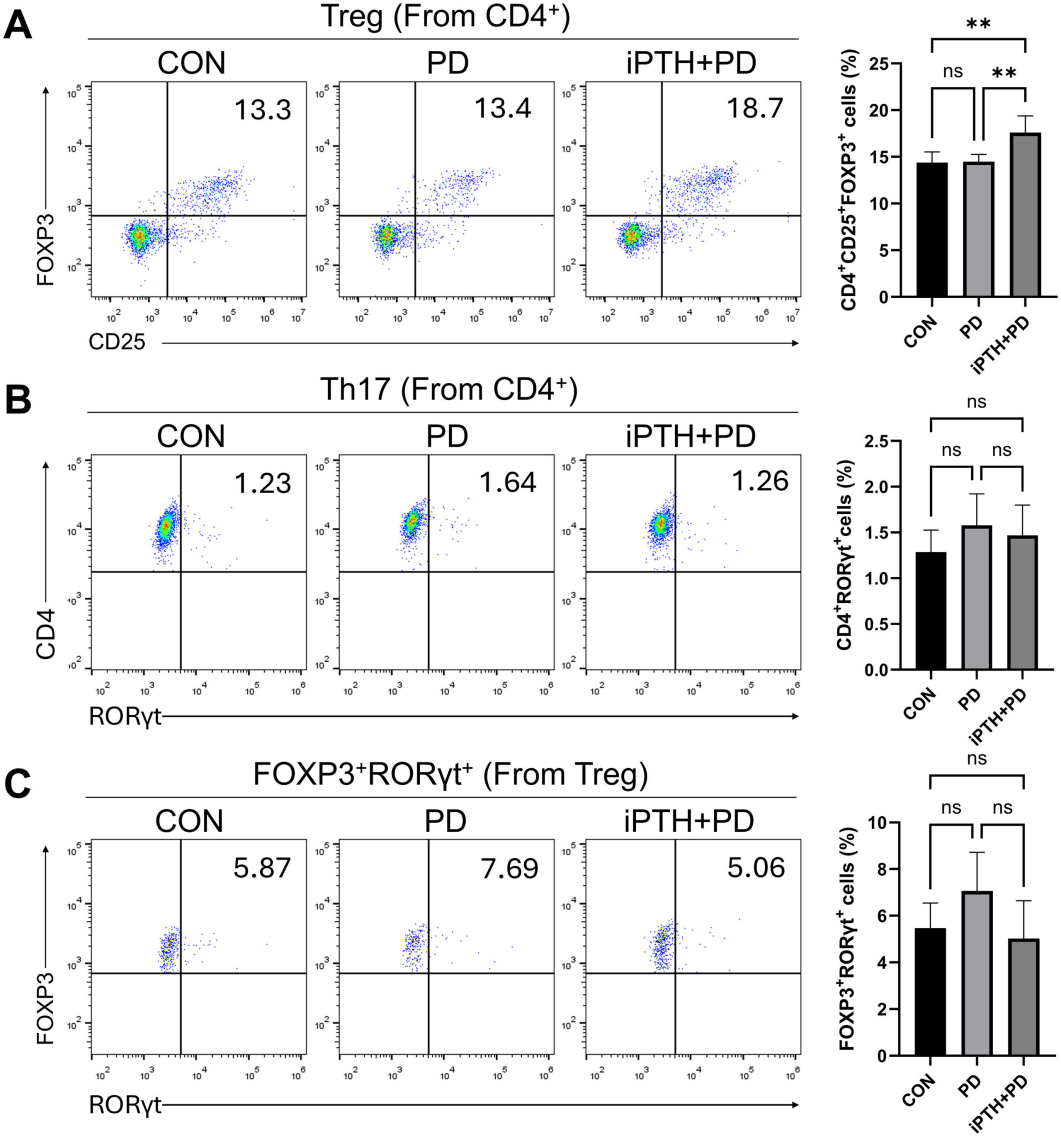
Systemic iPTH injection regulated the regulatory T cell (Treg) proportion in cervical lymph nodes (CLNs) in mice with periodontitis. **(A)** Representative flow cytometry plots and quantitative analysis of the frequency of CD4^+^CD25^+^FOXP3^+^ Tregs among CD4^+^ T cells. **(B)** Representative flow cytometry plots and quantitative analysis of the frequency of CD4^+^RORγt^+^ T helper cell 17 (Th17) among CD4^+^ T cells. **(C)** Representative flow cytometry plots and quantitative analysis of the frequency of FOXP3^+^RORγt^+^ cells among Tregs. Values were presented as mean ± SD. Significance was determined by one-way ANOVA with Tukey’s *post hoc* test (*n* = 6); ns, not significant; **p<0.01.

To further quantify the local frequencies of Treg and Th17 cells, we performed flow cytometry to assess their proportions among CD4^+^ T lymphocytes in the submandibular cervical lymph nodes. The results showed that the proportion of CD4^+^CD25^+^FOXP3^+^ Tregs remained comparable between the CON group and the PD group (*p* = 0.9903). In contrast, the iPTH+PD group showed a significantly increased proportion of Tregs relative to the PD group (*p=*0.0024) and the CON group (*p* = 0.0018) (**Figure 4A**).

Regarding Th17 cells, the proportion of CD4^+^RORγt^+^ cells was slightly elevated in the PD group compared with the CON group with no significance (*p=*0.2599). Meanwhile, the proportion of CD4^+^RORγt^+^ cells also did not differ significantly between the PD group and the iPTH+PD group (*p=*0.5750). The same was true between the CON and the iPTH+PD group (*p* = 0.8123) (**Figure 4B**).

Moreover, the proportion of FOXP3^+^RORγt^+^ cells among Tregs in the three groups exhibited similar outcomes as the proportion of CD4^+^RORγt^+^ cells, though did not reach statistical significance (CON vs. PD, *p* = 0.2599; CON vs. iPTH+PD, *p* = 0.5750; PD vs. iPTH+PD, *p* = 0.8123) (**Figure 4C**).

Collectively, these results indicated that iPTH elevated Treg proportion in CLNs.

### 3.6 Treg depletion abrogates the protective effect of iPTH on alveolar bone mass

**Figure 5:**
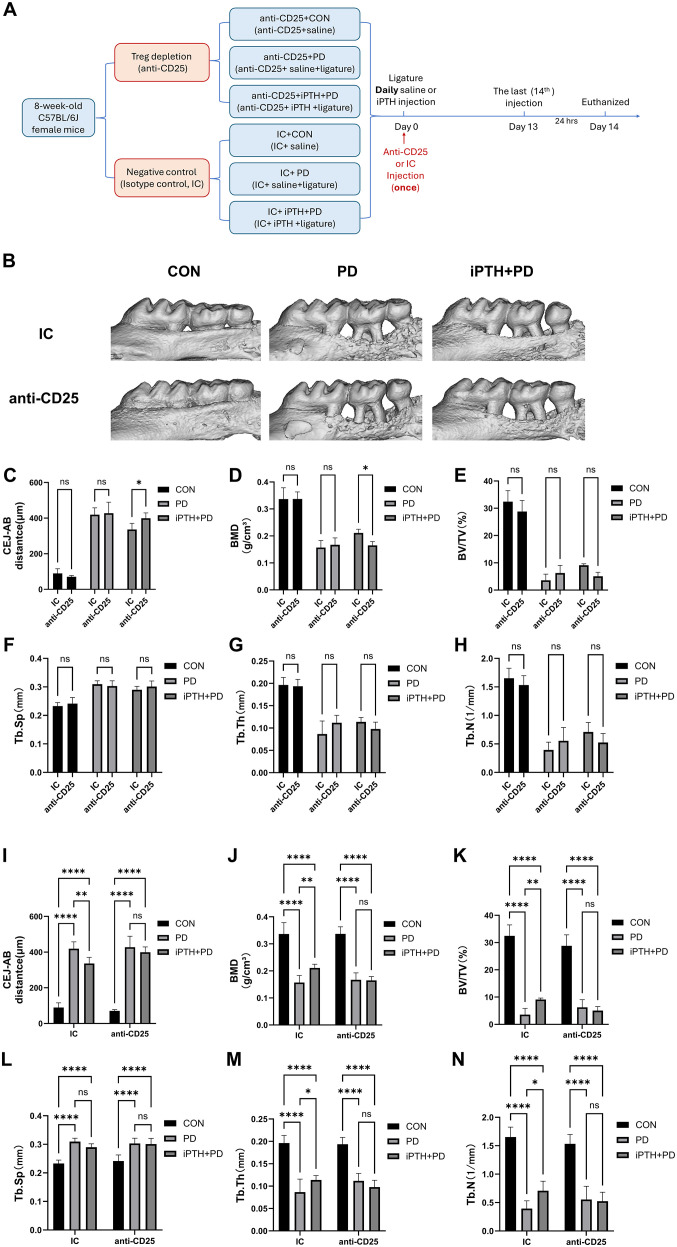
Anti-CD25-mediated Treg depletion reverses the protective effect of iPTH on alveolar bone mass in experimental periodontitis. **(A)** Schematic timeline of the experimental design for Treg depletion. **(B)** Representative buccal-view micro-CT 3D reconstructions of the alveolar bone from the CON, PD, and iPTH+PD groups, treated with either an anti-CD25 depleting antibody or an isotype control (IC). **(C–N)** Quantitative microarchitectural analysis of the alveolar bone, including the CEJ-ABC distance, BMD, BV/TV, Tb.Sp, Tb.Th, and Tb.N. Panel **(C–H)** compares the effects of anti-CD25 versus IC treatment across groups, while panel **(I–N)** compares the effects of periodontitis and iPTH treatment. Data are presented as mean ± SD. Significance was determined by two-way ANOVA with Tukey’s *post hoc* test (*n* = 5~6); ns, not significant, *p<0.05, **p<0.01, ****p<0.0001.

**Figure 6:**
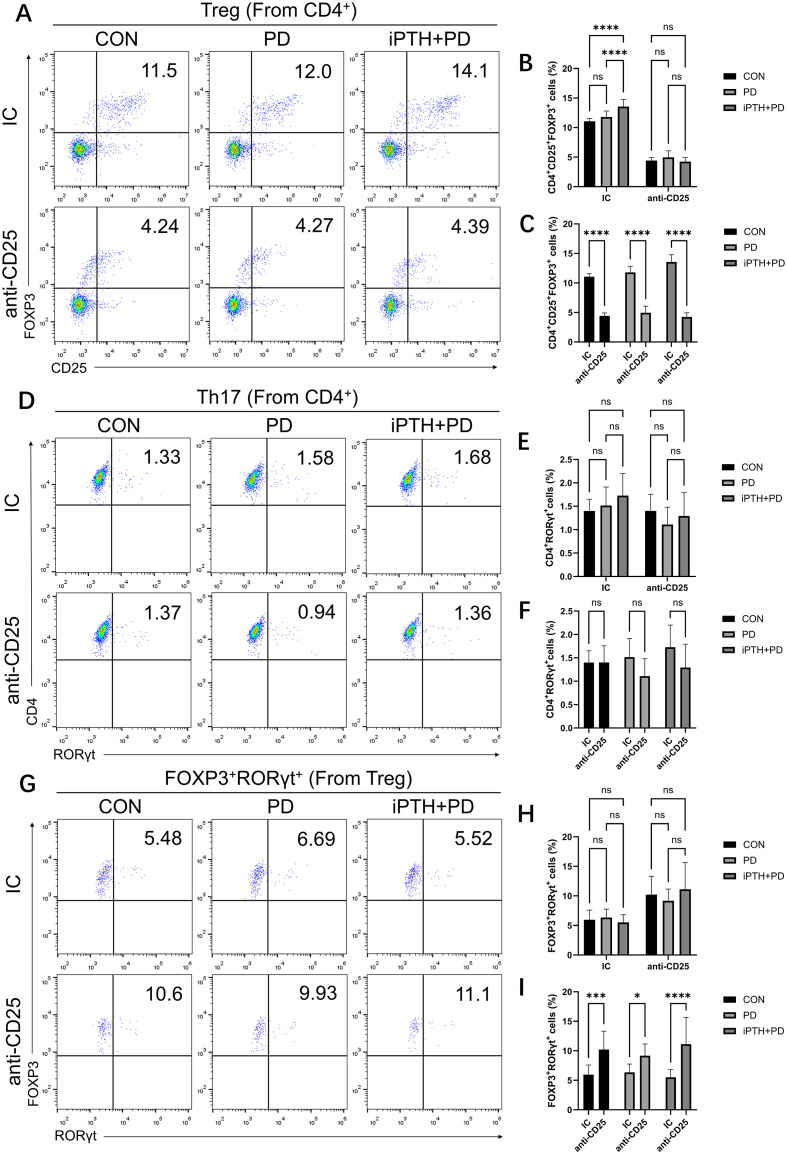
*In-vivo* Treg depletion by anti-CD25 disrupts the Treg and Th17 proportion in CLNs of mice with periodontitis. (ABC) Representative flow cytometry plots and quantitative analysis of the frequency of CD4^+^CD25^+^FOXP3^+^ Tregs among CD4^+^ T cells. (DEF) Representative flow cytometry plots and quantitative analysis of the frequency of CD4^+^RORγt^+^ Th17s among CD4^+^ T cells. (GHI) Representative flow cytometry plots and quantitative analysis of the frequency of FOXP3^+^RORγt^+^ cells among Tregs. Data are presented as mean ± SD. Significance was determined by two-way ANOVA with Tukey’s *post hoc* test (*n* = 12); ns, not significant, *p<0.05, ***p<0.001, ****p<0.0001.

To explore the role of Tregs in the mechanism by which iPTH inhibits alveolar bone loss in periodontitis, Tregs were depleted by anti-CD25 injection. The experimental design timeline was shown in **Figure 5A**. To assess the efficacy of Treg depletion, flow cytometry was performed. Significant reductions in Treg proportion were observed in the anti-CD25 groups relative to isotype control (IC) groups (*p<*0.0001). These results confirmed successful Treg depletion following anti-CD25 antibody administration (**Figure 6C**).

To confirm the effect of anti-CD25 and IC antibody on bone mass in each group, micro-CT was conducted and revealed no significant differences in all the parameters between the anti-CD25+CON group and the IC+CON group, as well as the anti-CD25+PD group and the IC+PD group (**Figures 5B–H**), indicating that anti-CD25 antibody administration basically did not affect alveolar bone mass in healthy and periodontitis mice. However, the CEJ-ABC distance was significantly higher (*p* = 0.0167) while BMD was significantly lower (*p* = 0.0158) in the anti-CD25+iPTH+PD group than in the IC+iPTH+PD group (**Figures 5C, D**).

To verify the effect of PD and iPTH under the condition of IC or anti-CD25 administration (**Figures 5B, I–N**), the micro-CT analysis showed that within the three IC-treated groups, the variation of alveolar bone height and trabecular indices was generally consistent with the former result. However, in the anti-CD25-treated groups, although the anti-CD25+PD group displayed a significantly decreased bone mass compared to the anti-CD25+CON group (all *p* < 0.0001), no significant differences in any of these parameters were observed between the anti-CD25+PD group and the anti-CD25+iPTH+PD group.

These results suggested that Treg depletion did not interfere with the bone mass in the CON and PD groups. On the contrary, Treg depletion abolished the ability of iPTH to ameliorate alveolar bone loss in periodontitis.

### 3.7 Treg depletion disrupts the regulation on Treg and Th17 proportion by iPTH in CLNs

As shown in **Figures 6A, B, D, E, G, H**, among the IC-treated groups, the proportions of Tregs, Th17 and FOXP3^+^RORγt^+^ Tregs shared similar outcomes with those previously observed in the CON, PD, and iPTH+PD groups. On the contrary, within the anti-CD25-treated groups, no significant variation on Treg, Th17 and FOXP3^+^RORγt^+^ proportion were observed.

What’s more, as shown in **Figure 6I**, the proportion of FOXP3^+^RORγt^+^ cells among Tregs in anti-CD25 treated groups were all significantly up-regulated compared to IC treated groups, while there was no significant change in Th17 (**Figure 6F**).

These results indicated that Treg depletion eliminated the effect of iPTH on Tregs but did not alter the proportion of Th17 cells. In addition, CD25 neutralization up-regulated the expression of RORγt in Tregs.

## 4 Discussion

Our study demonstrates that iPTH administration inhibits periodontitis-induced alveolar bone loss in mice, which is functionally dependent on the presence of Tregs.

### 4.1 iPTH administration ameliorates alveolar bone loss in periodontitis

Substantial evidence supports the role of iPTH/teriparatide in promoting maxillofacial bone repair. This includes applications in non-infectious healing, such as bone defect repair (23), tooth extraction socket healing (24, 25), and peri-implant osteointegration (26, 27), as well as in the treatment of infectious bone diseases like periodontitis (6, 28, 29), apical periodontitis (30), and bacteria-induced osteonecrosis (31). In both contexts, iPTH consistently enhances bone mass, with effects observable as early as one week (26). However, the underlying mechanisms appear context-dependent. In non-infectious situations, iPTH initially elevates both RANKL and OPG, boosting bone turnover early (24, 26), before favoring bone formation by lowering RANKL and elevating OPG at later stages (24). In contrast, in infectious conditions like periodontitis, iPTH ameliorates bone loss through inhibiting bone resorption (6, 28) and concurrently promoting bone formation (6, 29, 32). This is achieved by reducing osteoclast numbers and the RANKL/OPG ratio within 2–4 weeks (6, 29) and eliminating inflammatory cell infiltration (29, 30), findings that align with our results. We posit that this mechanistic divergence stems from iPTH’s ability to curb excessive osteoclast activity and inflammation in infectious states, meanwhile, a potential contribution of direct osteogenic effects cannot be excluded.

### 4.2 iPTH regulates the Treg/Th17 axis in periodontitis

A novel finding of our study is that the pharmacological action of iPTH in experimental periodontitis involves the regulation of the Treg/Th17 axis. The Treg/Th17 axis is operationally defined as the integrated net effect of Treg frequency (measured by flow cytometry and FOXP3 IHC) and Th17-associated effector activity (represented by tissue IL-17 protein and mRNA levels), rather than a strict quantitative ratio of the two subsets in the lesion.

Previous work by Yu et al. (15) and Li et al. (16) demonstrated that teriparatide up-regulates the Treg proportion in the bone marrow of healthy mice by activating TGF-β and IGF-1 signaling, which promotes Treg differentiation. Notably, Yu et al. (15) reported that iPTH did not increase Treg numbers in the spleen, intestine, or thymus of healthy mice. In this study, we observed an increased Treg proportion in CLNs and enhanced FOXP3 expression at the periodontal lesion after two weeks of iPTH treatment in periodontitis. We speculate that the systemic iPTH treatment promotes Treg differentiation, meanwhile, the inflammatory environment intensifies Treg chemotaxis, which synergistically enhance local Treg level at lesion site. Besides, CLNs serve as the regional immune organs for periodontitis, and their changes are expected to be more significant than those of distant organs such as the spleen or intestine.

While IL-17 signaling and Th17 cells have been implicated in the catabolic effects of continuous PTH administration (33) (34) and hyperparathyroidism (35), the direct impact of iPTH on IL-17 and Th17 had not been previously explored. We discovered that in gingival tissue, the IL-17 gene and protein level is significantly elevated in PD models but decreased after iPTH injection; while in CLNs, the proportion of CD4^+^RORγt^+^ Th17 cells displayed the similar trend without statistical significance. We suggest that this discrepancy revealed the difference between pathological change in gingiva and the immunological response in CLNs. IHC and PCR detects IL-17, the primary effector cytokine of Th17 cells. A marked increase in its protein and mRNA level in the gingival tissue directly confirms that Th17 cells were robustly activated in the local periodontal lesion. IL-17 induces keratinocytes and fibroblasts to produce RANKL and matrix metalloproteinases, which are closely associated with alveolar bone resorption and connective tissue destruction in periodontitis. In contrast, flow cytometry detects CD4^+^RORγt^+^ cells, reflecting the potential of CD4^+^ T cells differentiating toward the Th17 lineage. RORγt, as a key transcription factor for Th17 cells, indicates that the cells have acquired the identity of Th17 cells, but does not imply that they are actively secreting IL-17. The observed weak trend of an increased proportion of CD4^+^RORγt^+^ cells in CLNs suggests that periodontitis may induce the differentiation or expansion of Th17 cells in draining lymph nodes, however, this change may be less dramatic and less concentrated than the effector changes in the gingiva. Although a direct effect of iPTH on IL-17 conceivable, no studies to date have confirmed the expression of PTH1R on Th17 cells. Therefore, we hypothesize that iPTH may indirectly modulate Th17 function through other mediators.

While the Treg/Th17 axis is pivotal, emerging evidence suggests that the pathogenesis of periodontitis may involve FOXP3^+^IL-17^+^ or FOXP3^+^RORγt^+^ double-positive T cells. FOXP3^+^IL-17^+^ and FOXP3^+^RORγt^+^ T cells represent an intermediate or plastic state between Treg and Th17 lineages. In inflammation environment, Treg may develop into FOXP3^+^RORγt^+^ cells and lose suppressive function (36, 37). These double-positive cells were identified in human periodontitis lesions (38), associated with more severe clinical signs (38) and decrease with successful therapy (39). Besides, research showed elevated FOXP3 and RORγt in peri-implantitis (40), suggesting a Treg-Th17 hybrid state may be a feature of periodontal inflammation. Our flow cytometry data, showing a higher proportion of FOXP3^+^RORγt^+^ cells within the Treg population in the PD group compared to the control groups, supports this notion. This increase population is consistent with the elevated spatial proximity of FOXP3 and IL-17 observed via immunofluorescence in the PD group compared to control groups. What’s more, our study fills the gap of the regulation of FOXP3^+^RORγt^+^ T cells by iPTH in periodontitis condition. Both flow cytometry and immunofluorescence showed a decreased trend of gingival FOXP3/IL-17 co-expression or the proportion of FOXP3^+^RORγt^+^ T cells in CLNs after iPTH injection with periodontitis. Therefore, a key action of iPTH may be to enhance the stability of Tregs under inflammatory pressure, preventing their deviation toward a pro-inflammatory phenotype. Another explanation is that iPTH may promote the apoptosis or phenotypic shift of the double-positive T cells, which needs further research.

### 4.3 Treg depletion attenuates the protection of alveolar bone mass by iPTH

The crucial role of Tregs in mediating the bone-anabolic effects of iPTH is underscored by our finding that the anti-resorptive effect of iPTH in periodontitis is Treg-dependent. Given that alveolar bone resorption is the integrated pathological outcome reflecting the net balance of local inflammation and bone remodeling, the abrogation of iPTH’s bone-protective effect upon Treg depletion provides convincing evidence that Tregs are indispensable for iPTH’s therapeutic efficacy on periodontitis. On one hand, this is consistent with the work of Yu et al. (15), who showed that teriparatide fails to enhance bone mass in Treg-depleted healthy mice. Li et al. (16) further elucidated that Tregs mediate iPTH’s anabolic action by stimulating CD8^+^ T cells to produce Wnt10b, a potent osteogenic factor. This mechanism offers a plausible explanation for our observation that iPTH promotes the expression of BSP, a bone formation marker, in our periodontitis model. On the other hand, for inflammatory microenvironment like periodontitis, Tregs play a pivotal role in averting tissue damage resulting from uncontrolled pro-inflammatory responses by secreting anti-inflammatory factors like IL-10 and TGF-β, and expressing the inhibitory ligand CTLA-4, PD-1, etc. (41).

### 4.4 Limitations and future directions

The limitation of this study is that the mechanism through which systemic iPTH influences Treg/Th17 axis remains unrevealed. While Treg depletion experiment established the requirement of Tregs for the bone-protective effect of iPTH, the specific downstream effector pathways including inflammatory cytokines, osteoblast/osteoclast activity through which Tregs operate in this context warrant further investigation. While the present depletion experiment establishes the causal necessity of Tregs, future studies employing adoptive transfer of purified Tregs into Treg-depleted mice are warranted to test whether Tregs alone are sufficient to rescue the bone-protective effect. Besides, the effect of iPTH alone on immune response needs to be elucidated with full-factorial design. What’s more, due to limitation of experiment condition, we did not take IL-17A as a marker of Th17 for flow cytometry, leading to inaccuracy in measuring the proportion of functional Th17 populations. In addition, although iPTH demonstrated pronounced efficacy in both males and females, evidence indicates that the skeletal effects of PTH and its downstream signaling responses are modulated by sex and hormonal status (42, 43). Thus, conclusions drawn exclusively from the females may be difficult to applicate to male or mixed-sex populations, thereby limiting the general application of the findings.

Future research should aim to elucidate the precise signaling pathways by tissue-specific scRNA-seq through which systemic iPTH influences Treg/Th17 balance and Treg stability in both infectious and non-infectious bone repair contexts and its long-term influence on immune response. The intracellular staining of IL-17A should be adopted to quantify the functional Th17 cells. The quantification of Treg and Th17 within the inflamed tissue should be warranted. Sex-stratified analyses in conjunction with human cohort studies should be incorporated in future research. It would also be valuable to dissect whether local iPTH delivery recapitulates these immunomodulatory effects.

## 5 Conclusion

This study demonstrates that iPTH ameliorates periodontitis-induced alveolar bone loss through a Treg-dependent manner. Mechanistically, iPTH suppresses inflammation and shifts the RANKL/OPG ratio toward inhibition of osteoclastogenesis, while concurrently promoting bone formation. In addition, iPTH up-regulates Tregs and suppresses local IL-17 effector function., likely preventing their co-localization within gingival tissue. Treg depletion abolished the bone-protective effects of iPTH.

Collectively, our findings uncover a novel immunoregulatory role for iPTH, expanding its pharmacological profile and establishing this therapy as a potent modulator of alveolar bone homeostasis in periodontitis.

## Data Availability

The raw data supporting the conclusions of this article will be made available by the authors, without undue reservation.
