# Supplementary material for: Intermittent parathyroid hormone (1-34) ameliorates periodontitis via Treg-dependent immunomodulation of the Treg/Th17 axis
Source: Front Immunol. 2026 Jul 30;17:1862703. doi: 10.3389/fimmu.2026.1862703 (PMC13469451; doi:10.3389/fimmu.2026.1862703)
Supplement: Supplementary file 1 [file DataSheet1.pdf]

Supplementary Material

Gene Name	Forward Primer (5' to 3')	Reverse Primer (5' to 3')
Il6	TAGTCCTTCCTACCCCAATTTCC	TTGGTCCTTAGCCACTCCTTC
Il17a	TTTAACTCCCTTGGCGCAAAA	CTTTCCCTCCGCATTGACAC
Il10	TTAATAAGCTCCAAGACCAAGG	CATCATGTATGCTTCTATGCA
Tnfsf11	GCTGGGCCAAGATCTCTAAC	GTAGGTACGCTTCCCGATGT
Tnfrsf11b	CAGAGAAGCCACGCAAAAGTG	AGCTGTGTCTCCGTTTTATCCT
Gapdh	TGGAAGGGCTCATGACCACAG	GGGATGATGTTCTGGGCAGC

Supplementary Table.1 The sequences of primers used in this study.

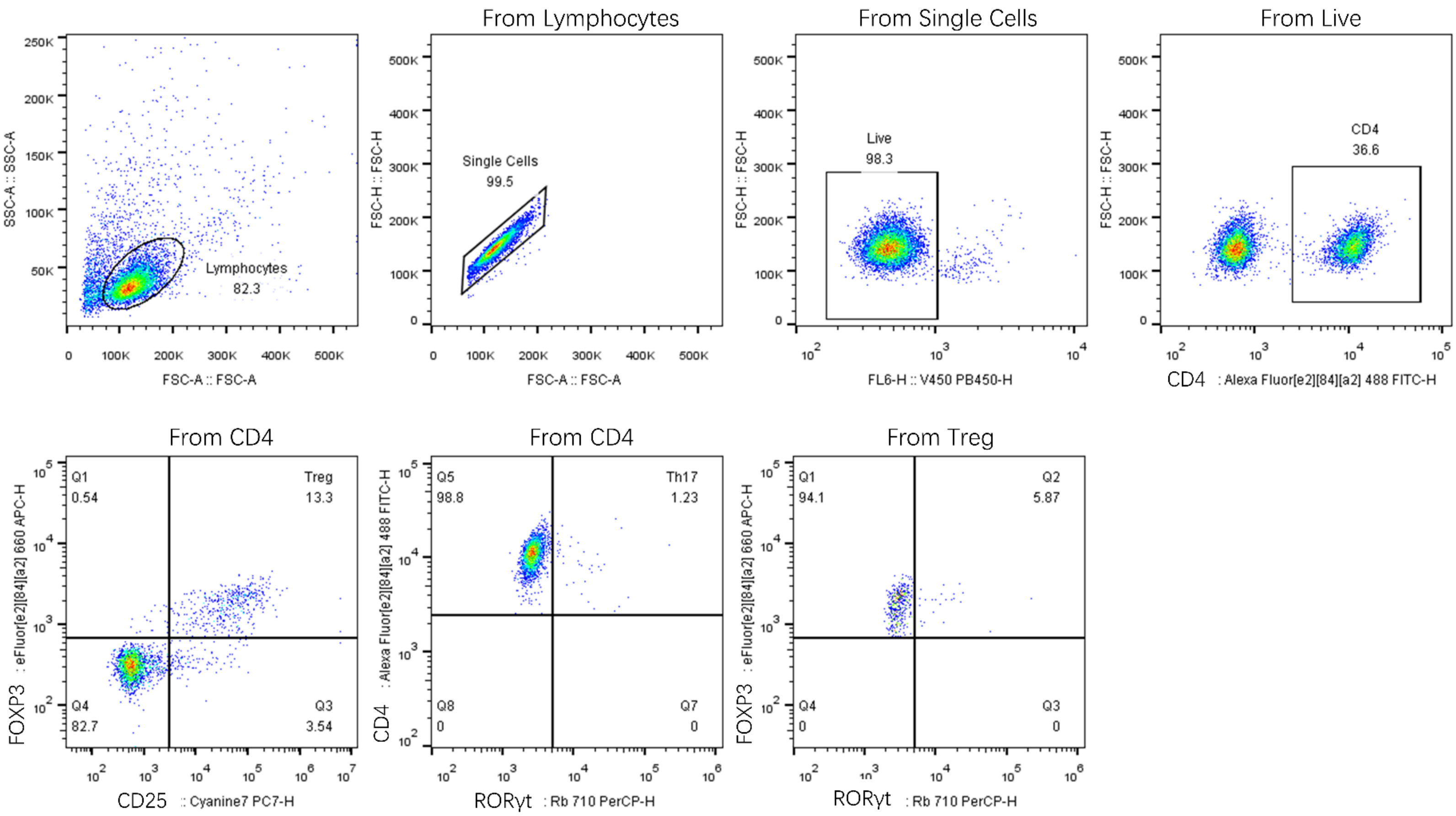

Supplementary Figure 1. The gating strategy for flow cytometry.
